# Do We Ever Need to Fix Clavicle Fractures in Adolescents?

**DOI:** 10.5704/MOJ.2311.006

**Published:** 2023-11

**Authors:** KBL Lim, RA Olandres, X Cheow, M Thng, NMHZ Teo, N Pereira, PXE Chan, NKL Lee

**Affiliations:** 1 Department of Orthopaedic Surgery, KK Women's and Children's Hospital, Singapore; 2 Division of Surgery, KK Women's and Children's Hospital, Singapore

**Keywords:** clavicle fracture, non-operative, displacement, shortening

## Abstract

**Introduction:**

Clavicle fractures in adults are increasingly being treated by surgical fixation following reports of symptomatic non-union, malunion and poor functional outcome with conservative treatment. This has led to a similar trend in the management of clavicle fractures in adolescents. This study aims to evaluate the outcome and complications of non-operatively treated clavicle fractures in adolescents.

**Materials and methods:**

This is a retrospective, single institution study on adolescents aged 13-17 years who sustained a closed, isolated clavicle fracture, between 19972015. Clinical records were reviewed for demographic information, injury mode, time to radiographic fracture union, time to re-attainment of full shoulder range of motion (ROM), and time to return to full activities and sports. Complications and fracture-related issues were recorded. Radiographs were analysed for fracture location, displacement and shortening.

**Results:**

A total of 115 patients (98 males, 17 females; mean age:13.9 ± 0.89 years) were included for study. 101 (88%) sustained a middle-third fracture while the remainder sustained a lateral-third fracture. A total of 96 (95%) of the middle-third fractures were displaced, and 12 (86%) of the lateral-third fractures were displaced. All displaced fractures in this study had shortening. Sports-related injuries and falls accounted for 68 (59%) and 34 (30%) of the cases respectively. Overall, the mean time to radiographic fracture union was 7.8 ± 4.35 weeks; there were no cases of non-union. Full shoulder ROM was re-attained in 6.6 ± 3.61 weeks, and full activities and sports was resumed in 11.4 ± 4.69 weeks. There were 5 cases of re-fracture and a single case of intermittent fracture site pain.

**Conclusion:**

Clavicle fractures in adolescents can and should be treated non-operatively in the first instance with the expectation of good outcomes in terms of time for fracture union, reattainment of shoulder full range of motion, and return to activities. Surgical stabilisation should be reserved for cases for which there is an absolute indication.

## Introduction

Historically, clavicle fractures have been treated non-operatively in both children and adults with satisfactory outcomes^1-3^. In the adolescent population, there is a small number of publications that similarly reports good outcomes following non-operative treatment of clavicle fractures^4,5^.

Non-union, significant malunion and other complications following clavicle fracture treatment are relatively uncommon^2,3^. Furthermore, a clavicular non-union has reportedly no significant effects on functional outcome or strength^6^. Displaced clavicle fractures that heal with 5mm or more shortening, while not uncommon, have also no clinical significance^7^.

Widely accepted `classical’ indications for surgical stabilisation of clavicle fractures for all age groups include polytrauma, open fracture, fracture with superior displacement - significant skin tenting with impending impalement, associated neurovascular compromise^1^, as well as an intention to return to competitive sports in the shortest possible time. In the absence of these factors, clavicle fractures are largely treated conservatively by simple immobilisation and rest. In many centres around the world today, this remains the mainstay of treatment for closed, uncomplicated clavicle fractures.

Several recent studies in adults, however, have reported poor outcomes in non-operatively treated clavicle fractures, particularly those with significant shortening and displacement^8-11^. A relatively high incidence of non-union has also led to poor outcomes^8,12^. The proponents of surgical fixation have argued that surgeon-based methods of clinical evaluation may be insensitive to the loss of shoulder strength and endurance^9^. In recent years therefore, there has been an increasing trend to treat displaced clavicle midshaft fractures in adults operatively^8,13^.

While the treatment of adolescent clavicle fractures was straightforward in the past – they were all managed non-operatively like in children– this has become increasingly controversial^14^, with a trend towards operative management as seen in adults^15^. An older teenager’s skeletal make-up is generally regarded to be more similar to that in adults than in young children, and therefore suboptimal outcomes reported with non-operative management are deemed applicable to adolescents. Many adolescents today are active in sports and participate at a professional level; they elect for surgical treatment in order to resume training and competition in the shortest possible time. Older children may achieve good results with non-operative treatment but have been reported to experience more pain and are likely to be dissatisfied with the cosmetic result and prolonged immobilisation^16^. As such, surgeons may have a lower threshold for managing adolescent clavicle fractures operatively.

Apart from the absolute or classic indications for surgical fixation, Yang *et al*^15^ have recommended that surgery should be considered for older male teens aged 15-19. Completely displaced midshaft clavicle fractures with shortening of greater than 2cm^17^, or 14-15%^18^, has been recommended as a relative indication for surgical fixation. Pandya *et al*^18^ recommend operative treatment for completely displaced fractures with no cortical contact, comminuted fractures, or those that contain a Z-shaped fragment; they further advocate that shortening alone in the absence of the above factors does not warrant surgical intervention. Overall, surgery for adolescent clavicle fractures has also been shown to be safe^19^ and achieve fracture union without exception^20^.

In our institution, closed, clavicle fractures in adolescents have been treated non-operatively over the years with good results, similar to that in several studies^4,5^. In the absence of the classic indications for surgery, we believe even significantly displaced and shortened clavicle shaft fractures in adolescents can be treated successfully with non-operative management.

In this review of non-operatively treated clavicle fractures in adolescents, our aims are to: (i) compare the outcomes of middle and lateral third clavicle fractures, (ii) report time to radiographic healing, time to re-attainment of full shoulder range of motion (ROM), and time to return to activities and sports, and (iii) identify complications and other issues. Our hypothesis is based on our experience over the years: that clavicle fractures in adolescents heal uneventfully without significant functional limitation and do not require surgical fixation.

In a recent systematic review of 19 reports on this subject, the “appropriate indications for non-operative care or relative indications for operative intervention are still unclear”^21^. We hope the results of this study can add to the literature and experience on the management of adolescent clavicle fractures.

## Materials and Methods

This single-institution, retrospective study was conducted at a tertiary children’s hospital. Patients’ medical records over a 19-year period (1997 – 2015) were reviewed. Healthy adolescents who sustained an isolated, closed clavicle fracture and were treated non-operatively, were included in this study. The Robinson Classification was used to classify the fractures. This study was conducted following Institutional Review Board approval.

Immobilisation was achieved using an arm sling, figure-of-eight bandage or collar and cuff. From patients’ medical records the following information was obtained: patient demographics, mechanism of injury, time to radiographic healing, time to re-attainment of full ROM of the affected shoulder, and time to return to sports. Radiographs were reviewed to determine fracture laterality and fracture site (middle, lateral or medial third), as well as displacement, shortening, comminution, and whether fracture union had been achieved. Patients were followed-up until there was radiographic evidence of fracture union, when full shoulder ROM was achieved and when full activities or sports was resumed. The time to resume full activities or sports was assessed based on the actual time reported by patients. If this was unavailable in the medical records, time given by physicians to excuse the patients from exercise and physical activity was used. The median follow-up period was 75 days. Complications and any other issues relating to symptoms, fracture union, and shoulder movement were also documented. Patients were excluded from the study if they had multiple fractures, incomplete or missing data, incomplete outpatient clinic follow-up, or other chronic medical conditions.

**Fig 1:**
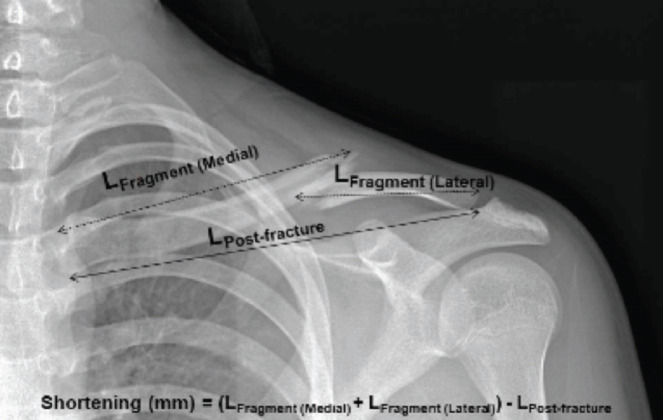
Method of measuring shortening of the midshaft clavicle fracture used in this study.

There is currently no validated nor standardised method of measuring clavicular shortening. Commonly used methods are unreliable and distinctly different from each other^22^. To account for shortening in angulated fractures, we devised a simple method of measuring clavicle shortening. The length of the medial fragment (LFragment (Medial)) and the length of the lateral fragment (LFragment (Lateral)), were first measured (Fig. 1). The length of the clavicle post-fracture (LPost-fracture) was then calculated by measuring the distance from the proximal (medial) end to the distal (lateral) end of the clavicle. Shortening was calculated by taking the sum of the two medial and lateral fragments, and then subtracting the length of the clavicle post-fracture. All measurements were expressed in millimetres (mm).

The data set was analysed using SPSS [IBM SPSS Statistics for Windows, Version 19.0. Armonk, NY: IBM Corp]. Continuous variables were summarised as descriptive statistics (mean, standard deviation, and percentage); categorical variables were expressed as number and percentage. Independent samples t-test was used to determine statistical significance in treatment outcomes between the middle third and distal third fracture groups. A p-value of less than 0.05 was considered as significant.

## Results

A total of 246 healthy patients, who sustained an isolated, closed clavicle fracture and were treated non-operatively, were screened for study eligibility and 115 met the criteria for inclusion. The remaining 131 were excluded because they had incomplete data or were lost to follow-up.

Data from 115 patients, aged 13-17 years inclusive, were included in the final analysis (Table I). The majority of fractures occurred in males (85%) and there were approximately equal left (48%) and right (52%) side clavicle fractures. Middle third clavicle fractures accounted for 88% of all the fractures, while the remaining were lateral third fractures (12%); there were no medial third fractures. The majority of these fractures were caused by sports-related injuries (59%), while the rest resulted from non-sports-related falls (30%), blunt injury (5%), road traffic accidents (5%), and assault (1%). Most clavicle fractures healed without complications (95%). However, 1 patient (1%) complained of intermittent fracture site pain and 5 patients (4%) sustained a refracture. In the subgroup of patients with refracture, there were 4 males and 1 female who were between 13 and 14 years of age. The mechanisms of refracture were fall (n=2) and sports-related (n=3). All but one of these refractures were displaced fractures, with a different time interval between the index fracture and refracture, occurring at 6, 8, 10, 11, 36 weeks from the index fracture. All healed uneventfully without the need for operative intervention.

**Table I:** Demographics and clinical characteristics of patient cohort

	n=115 (%)
Age, years	
Mean ± standard deviation	13.9 ± 0.89
Range	13 - 17
Gender	
Males	98 (85%)
Females	17 (15%)
Fracture Side	
Left	58 (48%)
Right	62 (52%)
Fracture Site	
Medial third (Type 1)	0 (0%)
Middle third (Type 2)	101 (88%)
Lateral third (Type 3)	14 (12%)
Mechanism of Injury	
Sports	68 (59%)
Falls	34 (30%)
Blunt Injury	6 (5%)
Road Traffic Accident (RTA)	6 (5%)
Assault	1 (2%)
Complications	
None	109 (95%)
Refracture	5 (4%)
Intermittent fracture site pain	1 (1%)

Note: Continuous data is expressed as mean ± standard deviation and range; categorical data is expressed as number and percentage.

**Fig 2:**
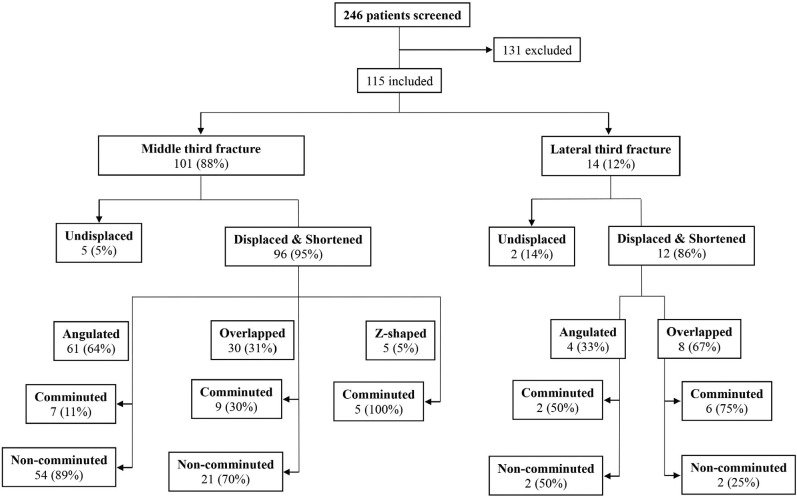
Patient selection process and fracture distribution flowchart.

Out of the 115 fractures, 108 (94%) were displaced, with a larger proportion of displaced fractures (89%) in the middle third fracture group (Fig. 2). All (100%) of the displaced fractures were also shortened, by 7.4% (middle third fractures) and 8.5% (lateral third fractures) (Table II). Comminution was present in 21 middle third fractures; 8 of the lateral third fractures were comminuted (Fig. 2).

**Table II:** Measurement of shortening in middle and lateral third displaced fractures

	Middle third	Lateral third
LFragment (Medial) (mm)	82.0 ± 11.49	103.6 ± 11.89
LFragment (Lateral) (mm)	63.0 ± 9.47	36.5 ± 16.56
LPost-fracture (mm)	134.1 ± 16.81	128.3 ± 11.35
Shortening (mm)	10.8 ± 6.72	11.9 ± 6.04
Shortening (%)	7.4 ± 4.34	8.5 ± 4.22

Note: Continuous data is expressed in mean ± standard deviation.

**Fig 3:**
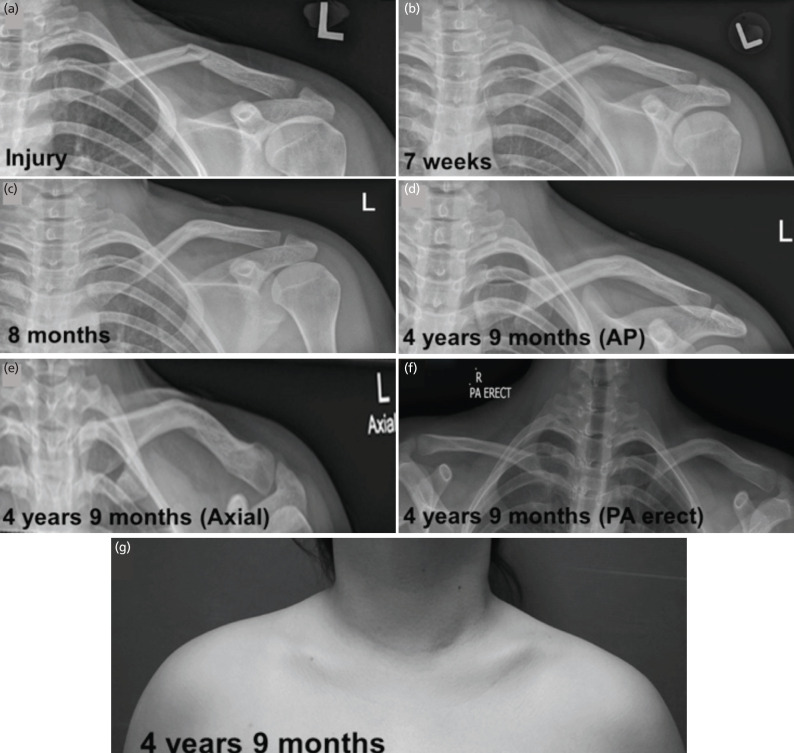
Radiographs and clinical photo of a 15-year-old female with a displaced midshaft left clavicle fracture. (a) First radiograph of injury, (b) AP radiograph at seven weeks, (c) AP radiograph at eight months, (d) AP radiograph at four years nine months, (e) Axial radiograph at four years nine months, (f) EOS radiograph at four years nine months, allowing comparison with non-fractured right clavicle, (g) clinical photo at four years nine months.

Overall, the mean time to radiographic healing was 7.8±4.35 weeks (range: 3 – 25), time to full ROM in the affected shoulder was 6.6±3.61 weeks (range: 2 – 24), and time to return to activity was 11.4±4.69 weeks (range: 3 – 30) from injury (Table III). When treatment outcomes were compared between the middle and lateral third clavicle fracture groups, no significant differences in the time to radiographic healing (7.8 ± 4.41 weeks vs 7.6 ± 4.07 weeks, p=0.867), time taken to re-attain full ROM (6.6±3.69 weeks vs 6.7±3.07 weeks, p=0.874), and time to return to activities (11.7±4.82 weeks vs 9.5±3.16 weeks, p=0.103) were observed. Lastly, all patients achieved fracture union and none of them required operative intervention. The radiographs and clinical photos of a typical adolescent with a displaced midshaft clavicle fracture, are shown in (Fig. 3).

**Table III:** Comparison of treatment outcomes between middle and lateral third clavicle fractures

	Overall	Middle third	Lateral third	p-value
Time to radiographic healing (weeks)	7.8 ± 4.35	7.8 ± 4.41	7.6 ± 4.07	0.867
Time to full range of motion (weeks)	6.6 ± 3.61	6.6 ± 3.69	6.7 ± 3.07	0.874
Time to return to activities (weeks)	11.4 ± 4.69	11.7 ± 4.82	9.5 ± 3.16	0.103

Note: Continuous data is expressed in mean ± standard deviation. Independent samples t-test was used to determine statistical significance. A p-value of less than 0.05 was considered as significant.

## Discussion

This report is the single largest series of adolescents (13 -17 years of age) with non-operatively treated clavicle fractures. The film (negative) radiographs of a large number of our earlier patients were retained by the patients and not archived in our institution; these patients were excluded in our analysis.

The results of this study are very positive. Fracture union was achieved without exception regardless of displacement, shortening or comminution. On average, these teenagers achieved fracture union within 8 weeks, regained full range of motion of the ipsilateral shoulder within 7 weeks, and resumed their normal activities and sports within 12 weeks. Patients can therefore be counselled accordingly at the outset. These time intervals are very similar to those reported by Vander Have *et al*^11^. There are several patients for whom the reported time intervals were not within the normal distribution, these are patients who deferred their clinic review and radiograph and were the outliers in this cohort.

Middle third fractures comprised 88% of this series, which is consistent with earlier published work^23,24^. There was no significant difference between middle and lateral third fractures with regard to time to radiographic fracture union, full shoulder ROM, and return to activities (Table III). The sample size of lateral third clavicle fractures in our study is much smaller than that of middle third fractures. Further work with a larger sample size would be needed to conclusively compare the outcomes of middle versus lateral third fractures; this is beyond the scope of this paper.

Good outcomes following the operative treatment of clavicle fractures in adults have led to a shift away from conservative management. Proponents of surgical treatment have also led us to believe that adolescents are more similar to adults physiologically than to children, and therefore the threshold for surgical fixation of clavicle fractures in adolescents should be lowered. While we agree with the absolute indications for surgery, we challenge the relative indications of fracture displacement, shortening and comminution^19^. Permanent shortening post-clavicle fracture has been reported to be relatively common, but of no clinical significance^7^.

The benefits of surgery must be weighed against the risks and potential complications. First, there are several reports of supraclavicular nerve injury and scar sensitivity^19^; surgical scars may be cosmetically unacceptable and especially so if they turn hypertrophic or form keloids. Second, a further operation may be necessary to remove implants, particularly if they are prominent or causing symptoms and pain^5,20^. This will invariably set the teenager back for an additional period of time. Following implant removal, there is also a risk of refracture at the previous implant sites. We would therefore like to lead the call to challenge the widely accepted relative indication for operative management of high-level adolescent athletes. Indeed, the fact that adolescent clavicle fractures heal more readily than those in adults is rarely contested. In this series, all patients were treated by non-operative means regardless of sporting activity, with good outcomes.

From the financial standpoint, operative treatment, regardless of the number of surgical procedures, will always incur a higher financial cost compared with conservative management. The routine fixation of midshaft clavicle fractures has shown not to be cost effective^25^. However, this statement is not all-encompassing since the minority of adolescents for whom non-operative management is no longer appropriate will be at higher risk with delayed surgery^26^.

In this series, there were five patients who sustained a refracture. In each case, the refracture was a result of a fall or sports-related injury in a young teenager aged 13 or 14 years. The refractures occurred over a wide time interval (6 to 36 weeks after the index fracture), but all healed uneventfully without surgery. We believe these refractures to be a direct result of definite trauma in active, young teenagers, rather than low-energy injuries in osteopenic or abnormal bone.

A single patient reported intermittent fracture site pain. We were unable to obtain more detailed information about his pain from his records and were unable to contact him to enquire further about his symptoms. No other patient reported fracture site pain once fracture union was achieved. We do not believe this single case of intermittent post-fracture pain to be significant.

Limitations of this study: This retrospective study has several limitations. The amount of fracture shortening, known to be an important determinant of suboptimal outcomes if greater than 2cm^27^ was not measured and recorded in the case records. Shortening was not measured in our institution as surgeons did not find a reliable method they could apply, and were also of the view that any such measurement was highly variable and lacked standardisation. Skeletal age, which may indicate growth and recovery problems when differing from chronological age, as well as hand dominance, which has shown to affect function^28^ and may predispose unfavourable outcomes in conservative treatment^29^, were also not measured nor recorded in the case records.

In the course of the study period, the type and duration of fracture immobilisation varied according to surgeon preference, as did the frequency and interval of outpatient follow-up visits. We believe these variations in the treatment regimens would have not significantly affected the outcomes of non-operative treatment of these fractures.

In this study, there were many more males than females, and similarly, many more middle third fractures than lateral third fractures. The sizes of the subgroups were therefore very different and may not make for an ideal comparison. There were only 21 comminuted midshaft fractures, and therefore this sample size is insufficient to support the broad conclusion that comminuted fractures should be managed non-operatively. While we could have reported on a more homogenous group comprising only adolescent males with a midshaft clavicle fracture, we decided to include females and lateral third clavicle fractures, in order to provide an overall perspective in a tertiary clinical practice setting.

Assessment of patients during the recovery phase was done largely based on subjective information and gross clinical examination, rather than objective strength testing. Evaluation with validated instruments such as the Disabilities of the Arm, Shoulder and Hand (DASH), or simple shoulder test (SST) for patient-reported outcomes. Alternatively, the Constant Score (CS), with different weightings for pain and activities of daily living reported by patient, and range of movement and strength measured objectively by a clinical observer, would have provided more valuable outcome measures. Data on approval or disapproval of cosmesis after non-operative management was not available.

While the majority of the patients in our study sustained their injuries through sports, the clinical records did not contain enough detail for us to stratify the patients according to the level of demand of their sporting activities. It would be interesting to see if the results in our study would hold true especially for high demand adolescent athletes. Nevertheless, we hope our paper would lead the call for further work to be done to look into the non-operative treatment outcomes amongst adolescent athletes of varying levels of competitiveness.

Relevance of this study: This study provides the evidence that middle and lateral third clavicle fractures in adolescents can be managed non-operatively with very good outcomes. Both patients and surgeons can be convinced that non-operative treatment does yield good results and should always be considered as a first option. We hope this report removes some controversy in the management of uncomplicated clavicle fractures in adolescents.

## Conclusion

Closed clavicle fractures in adolescents (aged 13-17) should be treated non-operatively, with the expectation of very good outcomes in terms of time to fracture union, recovery of full range of motion, and return to activities. Operative treatment should be reserved for cases where there is an absolute indication for surgery – such as polytrauma, open fracture, fracture with superior displacement and an intention to return to competitive sports in the shortest possible time. The benefits of surgical fixation must be weighed against its potential risks and complications, as well as the need for a second procedure for implant removal.
